# Characterization of a *Straboviridae* phage vB_AbaM-SHI and its inhibition effect on biofilms of *Acinetobacter baumannii*

**DOI:** 10.3389/fcimb.2024.1351993

**Published:** 2024-03-08

**Authors:** Liming Jiang, Qian Xu, Ying Wu, Xianglian Zhou, Zhu Chen, Qiangming Sun, Jinsheng Wen

**Affiliations:** ^1^ School of Basic Medical Sciences, Health Science Center, Ningbo University, Ningbo, Zhejiang, China; ^2^ Department of Blood Transfusion, Hubei No. 3 People’s Hospital of Jianghan University, Wuhan, Hubei, China; ^3^ Department of Rheumatology Immunology, The First People’s Hospital of Hefei, Hefei, Anhui, China; ^4^ Department of Laboratory, Ningbo No.2 Hospital, Ningbo, Zhejiang, China; ^5^ National Kunming High-level Biosafety Primate Research Center, Institute of Medical Biology, Chinese Academy of Medical Sciences and Peking Union Medical College, Kunming, Yunnan, China

**Keywords:** *Acinetobacter baumannii*, phage, bacterial biofilm, antibiotic, inhibition

## Abstract

*Acinetobacter baumannii* (*A. baumannii*) is a popular clinical pathogen worldwide. Biofilm-associated antibiotic-resistant *A. baumannii* infection poses a great threat to human health. Bacteria in biofilms are highly resistant to antibiotics and disinfectants. Furthermore, inhibition or eradication of biofilms in husbandry, the food industry and clinics are almost impossible. Phages can move across the biofilm matrix and promote antibiotic penetration. In the present study, a lytic *A. baumannii* phage vB_AbaM-SHI, belonging to family *Straboviridae*, was isolated from sauce chop factory drain outlet in Wuxi, China. The DNA genome consists of 44,180 bp which contain 93 open reading frames, and genes encoding products morphogenesis are located at the end of the genome. The amino acid sequence of vB_AbaM-SHI endolysin is different from those of previously reported *A. baumannii* phages in NCBI. Phage vB_AbaM-SHI endolysin has two additional β strands due to the replacement of a lysine (K) (in KU510289.1, NC_041857.1, JX976549.1 and MH853786.1) with an arginine (R) (SHI) at position 21 of *A. baumannii* phage endolysin. Spot test showed that phage vB_AbaM-SHI is able to lyse some antibiotic-resistant bacteria, such as *A. baumannii* (SL, SL1, and SG strains) and *E. coli* BL21 strain. Additionally, phage vB_AbaM-SHI independently killed bacteria and inhibited bacterial biofilm formation, and synergistically exerted strong antibacterial effects with antibiotics. This study provided a new perspective into the potential application value of phage vB_AbaM-SHI as an antimicrobial agent.

## 1 Introduction

*Acinetobacter baumannii* (*A. baumannii*) is a gram-negative opportunistic pathogen that mainly causes various infections, such as pneumonia, urinary tract infection, ventilator-acquired pneumonia and bacteremia. This bacterium was widely distributed in water, soil, food, activated sludge, and on human body (Antunes et al., 2014; Potron et al., 2015; Bello-López et al., 2020). With the widespread abuse and misuse of antibiotics, multidrug-resistant *A. baumannii* has been isolated from patients in hospital since the 1990s (Zheng et al., 2013; Ahmed et al., 2016). Unfortunately, more than 80% *A. baumannii* possess multidrug resistance (MDR) to antibiotics (such as ampicillin, amoxicillin, clavulanate, cefotaxime, ceftriaxone, aztreonam, ertapenem, trimethoprim, fosfomycin, tetracycline and colistin, etc) due to the presence of multiple genetic elements encoding antimicrobial substance (Lee et al., 2011; Pendleton et al., 2013; Wannigama et al., 2019; Srisakul et al., 2022). Moreover, *A. baumannii* has a strong ability to form biofilm, which exacerbates antibiotic resistance in surgical devices, catheters and medical implants (Gayoso et al., 2014). The World Health Organization classified *A. baumannii* as priority pathogen in 2017 because of the lack of new therapies, which highlights the urgent need for the development of novel targeted therapies (Gauba and Rahman, 2023).

Phage endolysin is a cell wall hydrolase that is synthesized by phage at the later stage of phage infection, it has the ability to selectively kill bacteria when applied from outside the cell (Fischetti, 2008; Ghose and Euler, 2020; Cui et al., 2023; Nguyen et al., 2023). The majority of endolysins with reported intrinsic antibacterial activity have a C-terminal amphipathic helix (Gutiérrez and Briers, 2021) and the phage endolysin of gram-negative bacteria is a small globular protein with a single enzyme activity domain (Briers et al., 2007). Given the broad host spectrum, phage endolysins have significant advantages in treating bacterial infections. Wang et al., reported that TSPphg lysin was a potential antimicrobial agent which could inhibit both gram-negative (*Salmonella paratyphi* B, *Escherichia coli* O157 and *Klebsiella pneumoniae*) and gram-positive (*Bacillus subtilis* and *Staphylococcus aureus*) bacteria growth (Wang et al., 2020a; Shein et al., 2023).

Bacterial biofilms are composed of bacteria embedded in a self-produced extracellular matrix on nonbiotic and biotic surfaces (Hall-Stoodley et al., 2004; Jakubovics et al., 2021). Bacteria in biofilms are highly resistant to antibiotics and disinfectants (Venkatesan et al., 2015; Oliveira et al., 2019), and the inhibition or eradication of biofilms in husbandry, the food industry and clinics were almost impossible (Penesyan et al., 2015; Kim et al., 2018). Although disinfectants and antibiotics play the important roles in controlling bacterial infections, the emergence of drug-resistant bacteria has changed the dominance of antibiotics (Chapman and Gunter, 2018).

As a virus, phages have the ability to lyse their bacterial hosts (Gutiérrez et al., 2016; Sharma et al., 2017). Phages can also inhibit the formation of biofilms of *E. coli, Listeria monocytogenes, Salmonella*, and *Pseudomonas aeruginosa* (de Ornellas Dutka Garcia et al., 2017; Gong and Jiang, 2017; Chegini et al., 2020; Tian et al., 2021). In particular, phages were harmless to humans and safe in the clinic and food products (Ahn et al., 2013). Therefore, phages are expected to become a good alternative or complement to antibiotics (Endersen et al., 2014; Shein et al., 2023).

In the present study, a lytic *A. baumannii* phage vB_AbaM-SHI was isolated from a sauce chop factory drain outlet in Wuxi, China. The genomic features of phage vB_AbaM-SHI were characterized. The capacity of phage vB_AbaM-SHI to lyse *A. baumannii* and inhibit the biofilm formation of *A. baumannii* was explored. Herein, we aimed to determine whether phage vB_AbaM-SHI represents a potential therapeutic agent for multidrug-resistant bacteria *A. baumannii*.

## 2 Materials and methods

### 2.1 *A. baumannii* strain and cultivation

*A. baumannii* SL was obtained from the China Microbial Culture Preservation Center (CMCPC) and grown aerobically in brain-heart infusion (BHI) broth culture or on BHI plates (Difco, MI, USA) at 37°C, and used as a host for phage isolation. *A. baumannii* cpx isolates (SL1, SJ9, SA5, SA3, SG, SH) were obtained from department of clinical laboratory, the first people’s hospital of Yunnan province, China. BHI soft agar containing 0.45% or 0.5% (W/V) agar was used for phage plaque confirmation. *A. baumannii* stock culture was stored at −80°C with 20% (V/V) glycerol.

### 2.2 Phage vB_AbaM-SHI isolation and purification

An *A. baumannii*-targeting phage was isolated from sauce chop factory drain outlet samples in Wuxi, China. Isolation of phage was performed as described in previous study (Park et al., 2012). In brief, 10 g of the sauce chop factory drain outlet sample were mixed with 20 mL of sterile Phosphate Buffered Saline (PBS) and incubated for 2 h at room temperature with shaking at 200 rpm. The sample was centrifuged at 5, 000×g for 15 min, followed by filtration with a 0.22 μm filter membrane. Ten milliliters of the filtrate were added to 40 mL of BHI broth containing a 1:100 *A. baumannii* SL overnight culture and incubated for 48 h. Afterwards, the culture was centrifuged at 8, 000×g for 15 min, followed by filtration with a 0.22 µm filter membrane. The filtrate was serially diluted (10-fold) and mixed with 6 mL of molten 0.5% BHI soft agar containing *A. baumannii* SL (2×10^8^ cfu/mL), and then spread onto BHI plate. 24 hours later, the phage plaque was observed by naked eye and a single plaque was selected for phage purification, and the process was repeated for three times.

### 2.3 Evaluation of the sensitivity of the phage to physicochemical factors

The dilution (1 × 10^8^ pfu mL^-1^) was performed by referring to the phage vB_AbaM-SHI titer in the storage solution with BHI broth culture. For the thermal stability experiment, one milliliter of diluted phage vB_AbaM-SHI was incubated for 1 h at the temperature of 4°C, 25°C, 37°C, 42°C, 50°C, 60°C or 90°C, respectively. As for the pH stability experiment, 0.99 mL of buffer at pH 3, 4, 5, 6, 7, 8, 9, 10 or 11 (citrate buffer, 50 mmol/L, pH 3, pH 4 or pH 5; phosphate buffer, 50 mmol/L, pH 6, pH 7 or pH 8; Tris-HCl buffer, 50 mmol/L, pH 9; sodium carbonate buffer, 50 mmol/L, pH 10 or pH 11) was mixed with phage vB_AbaM-SHI (1 × 10^8^ pfu mL^-1^) in a 1.5 mL sterile centrifuge tube and incubated at 20°C for 1 h. As control, we did a complementary experiment of phage killing among the different buffers with the same pH 7. The multiplicity of infection (MOI) indicates the ratio of phage vB_AbaM-SHI to *A. baumannii* SL during the initial infection. Phage vB_AbaM-SHI stocks were added to the *A. baumannii* SL culture medium at MOIs of 100, 10, 1, 0.1, 0.01, 0.001 or 0.0001, and then cultivated at 37°C for 12 h. *A. baumannii* SL culture were centrifuged at 9, 000 × g for 10 min and the supernatant was filtered through a 0.22 µm filter membrane. The phage titer was determined using the double layer agar method as described previously (Park et al., 2012) and expressed as plaque-forming unit (pfu) mL^-1^. The experiment was repeated for three times.

### 2.4 Adsorption rate and one-step growth curve

To measure the adsorption rate of phage on host cell, phage vB_AbaM-SHI (1×10^8^ pfu mL^-1^) and logarithmic phase *A. baumannii* SL were mixed at a MOI of 0.1 and incubated at 37°C. The phage titer in the supernatant was measured at 0, 2, 4, 6, 8, 10, 15, 20 and 30 min after phage infection. The one-step growth curve of phage vB_AbaM-SHI was carried out as follows. Briefly, 10 mL of exponential phase *A. baumannii* SL culture was centrifuged (5, 000 g, 4 min, 37°C) and the cell pellet was resuspended in 20 mL of fresh BHI to obtain an OD_600_ of 1. Next, 20 mL of phage vB_AbaM-SHI was added to the bacteria to reach a MOI of 1 and allowed to adsorb for 10 min at 37°C. The mixture was centrifuged at 5, 000 × g for 4 min at 37°C, and the pellet was resuspended in 10 mL of fresh BHI. Samples were taken up to 90 h, after which the supernatants were plated on BHI agar to determine the phage titer.

### 2.5 Transmission electron microscopy

The Phage vB_AbaM-SHI particles were analyzed using transmission electron microscopy (TEM). Dilutions of the phage vB_AbaM-SHI stock (4×10^8^ to 4×10^9^ pfu mL^-1^) were deposited on copper grids with carbon-coated formvar films for 10 min and negatively stained with 2% uranyl acetate (pH 4.0) for 2 min. The phage particles were imaged using a Philips EM 300 electron microscope operated at 80 kV at Wuxi Jiangnan University (China).

### 2.6 Phage vB_AbaM-SHI genomic DNA extraction

Firstly, the purified phage vB_AbaM-SHI was concentrated through a 10 kDa filter (approximately 10^9^ to 10^10^ pfu mL^-1^). Then, the phage vB_AbaM-SHI genomic DNA was extracted using a Takara Minibest Viral RNA/DNA Extraction Kit (Cat#9766). Finally, purified phage vB_AbaM-SHI genomic DNA was treated with RNase at 37°C for 1 h. The restriction endonucleases Hind III, EcoRI, Not I and Xhol I (Takara) were used to digest the phage genomic DNA at 37°C for 1 h.

### 2.7 Complete genome sequence and bioinformatics analyses of vB_AbaM-SHI

The phage vB_AbaM-SHI genome was sequenced using the Illumina HiSeq platform (Sangon Biotech, China). The high-quality original sequencing data was assembled with *de novo* assembler software (Roche, Mannheim, Germany) at Sangon Biotech, China and analyzed with SPAdes software. Phage vB_AbaM-SHI genome annotation was carried out using Prokka 1.13.7. PCR amplification was performed based on a pair of primers targeting the start and end positions of the phage genome, respectively, to determine whether the phage genome is linear or circular. The complete genome sequence of phage vB_AbaM-SHI was deposited into the NCBI GenBank database (https://www.ncbi.nlm.nih.gov/nuccore) (GenBank Accession no: ON480525). The protein sequences of phage vB_AbaM-SHI were obtained using functional annotation. The Basic Local Alignment Search Tool (BLAST) was used to compare phage genome sequences from multiple databases including COG, TrEMBL, KOG, PFAM, CDD, NT, NR and SwissProt. Comparative genomic analysis among *Acinetobacter* phage IME-AB2 (NC_041857.1), *Acinetobacter* phage vB_AbaM_IME285 (MH853786.1) and *Acinetobacter* phage vB_AbaM-SHI were performed using the Easyfig 2.2.5 visualization tool (https://mjsull.github.io/Easyfig/). Phage vB_AbaM-SHI was identified and classified according to the International Committee on Taxonomy of Viruses (ICTV) (Adriaenssens and Brister, 2017).

### 2.8 Phage lysis and antibiotic sensitivity experiments of *A. baumannii*

The bacterial spectrum of phage vB_AbaM-SHI was determined by the spot test method. The reference bacterial strains (*S. aureus* cpx, *Salmonella paratyphi* A NA3, *Escherichia coli* BL21, and *A. baumannii* cpx) were tested for the susceptibility to phage vB_AbaM-SHI. 200 ml of bacteria (10^9^ cfu mL^-1^) were mixed with 5 ml of molten 0.5% BHI soft agar and spread onto BHI 1.8% (w/w) agar plates [colony-forming unit (cfu)]. Five minutes later, one drop of phage vB_AbaM-SHI suspension was added to the plate and incubated at 37°C for 18 h. The susceptibility of *A. baumannii* strain to five antibiotics (ampicillin, ciprofloxacin, levofloxacin, tobramycin and colistin) was tested using the minimal inhibitory concentration (MIC) method of serial broth microdilution.

### 2.9 The effects of phage vB_AbaM-SHI and kanamycin sulfate on biofilms formation

The polylysine-coated cell slides were placed to each well of 6-well plates for scanning electron analysis. The *A. baumannii* SL stock was inoculated into 100 mL of BHI broth culture at a ratio of 4:1000. Thereafter, 2 mL of the bacterial culture was inoculated into each well of 24-well plates. In one experiment, the test wells were added with phage vB_AbaM-SHI (MOI = 0.1), kanamycin sulfate (10 µg mL^-1^), or a mixture of kanamycin sulfate and phage vB_AbaM-SHI, whereas the wells receiving only BHI broth culture were used as control. In another experiment, *A. baumannii* SL inoculated in 24-well plate was cultivated for 12 h, followed by addition of phage vB_AbaM-SHI, kanamycin sulfate, or a mixture of kanamycin sulfate and phage vB_AbaM-SHI. Twelve hours later, the mount of *A. baumannii* SL in the broth culture was measured using the plate counting method. In addition, the cell slides were washed twice with PBS, fixed with pre-cold 2.5% glutaraldehyde for 2 h, and dehydrated with a gradient of ethanol solutions (15%, 30%, 40%, 50%, 70%, and 100%, respectively) for 15 min. Finally, the bacterial morphology was analyzed using a scanning electron microscope (SEM) after overnight air dry as described previously (Jiang et al., 2021b).

The *A. baumannii* SL stock was inoculated into BHI broth culture at a proportion of 1:250 and cultivated overnight. The diluted bacterial culture (200-fold) was added to each well of 96-well plates (200 µL/well). In one experiment, phage vB_AbaM-SHI (MOI = 0.1), kanamycin sulfate (10 µg mL^-1^), or a mixture of kanamycin sulfate and phage vB_AbaM-SHI was added to the test well. The control wells were added with only broth culture. The bacteria were then cultivated at 37°C for 24 h. In another experiment, *A. baumannii* SL was first cultivated for 12 h, and then added with phage vB_AbaM-SHI, kanamycin sulfate, or a mixture of two components. The wells given with only broth culture worked as control. The bacteria were then cultivated at 37°C for 24 h. The OD_600_ of each well in the plate was read using microplate reader. The supernatant in the plates was discarded and the plates were washed twice with PBS and fixed with 99% methanol for 10 min. After discarding the methanol, 2% crystal violet solution was added to each well of the plate and incubated for 10 min. The OD_570_ value of each was measured using microplate reader after rinsing the plate with water.

To determine the ability of phage vB_AbaM-SHI to inhibit the formation of *A. baumannii* SL biofilms, the cell slides were placed into each well of 12-well plates. *A. baumannii* SL stock was inoculated into 200 mL of BHI broth culture at a ratio of 4:1000. 1 mL of *A. baumannii* SL culture was inoculated into each well 12-well plates. The test wells were added with phage vB_AbaM-SHI (MOI = 1) whereas control wells were given with only broth culture. After a 36-h incubation at 37°C, the supernatants in the plates was discarded and the plates were washed twice with PBS and fixed with 99% methanol for 10 min. After discarding the methanol, 2% crystal violet solution was added to each well of the plate and incubated for 10 min. The morphology and amount of biofilms were analyzed using epifluorescence microscopy as described previously (Duan et al., 2023). Three repeated tests were performed. The experiment was repeated three times.

### 2.10 Tertiary structure prediction and phylogenetic analysis of *A. baumannii* phage endolysins

The endolysin amino acid sequences of *A. baumannii* phage vB_AbaM-SHI, and the other *A. baumannii* phages (AJG41880.1, AJG41879.1, KU510289.1, AJG41878.1, AJG41877.1, NC_041857.1, ASN73403.1, ASN73457.1, MH853786.1, AJG41885.1, AJG41884.1, ALJ97637.1, ASN73506.1, AJG41883.1, ADG35978.1, YP_004009630.1, AJG41882.1, AXF40585.1, YP_006488979.1, AXY82632.1, AJT61420.1, AWY10427.1, AJT61311.1, YP_009055463.1, QBY34658.1, QBY34659.1, AID17956.1, QBY34657.1 and QBY34660.1) were obtained from the NCBI database (https://www.ncbi.nlm.nih.gov/) and shown in **Table 1**. Tertiary structure homology modeling of *A. baumannii* phage endolysins was performed using the SWISS-MODEL online suite and the applied modeling templates were shown in **Table 2**. The phylogenetic analysis of *A. baumannii* phage endolysins was performed with the Neighbor-Joining program of Molecular Evolutionary Genetics Analysis (MEGA7.0) from multiple alignments done with MUSCLE (MEGA7.0) (Jiang et al., 2018). The number on the branch represents the bootstrap value for 1000 replicates. The amino acid specificity of endolysin was analyzed using BioEdit software.

**Table 1:** Characteristics of *A. baumannii* phage endolysins.

References of phage endolysins	Lengths of amino acid sequences
QBY34658.1, QBY34659.1, QBY34660.1 and QBY34657.1	145
KU510289.1, NC_041857.1, MH853786.1 and vB_AbaM-SHI	170
AJG41880.1, AJG41879.1, AJG41878.1 and AJG41877.1	180
AJT61420.1, AJT61311.1, YP_009055463.1 and AID17956.1	184
ALJ97637.1, ASN73403.1, ASN73457.1 and ASN73506.1	185
AXF40585.1, YP_006488979.1, AWY10427.1 and AXY82632.1	188
ADG35978.1 and YP_004009630.1	190
AJG41885.1, AJG41884.1, AJG41883.1 and AJG41882.1	256

**Table 2:** Modeling templates for predicting the tertiary structure of *A. baumannii* phage endolysins.

References of phage endolysins	Modeling templates
ADG35978.1, YP_004009630.1, AXF40585.1, YP_006488979.1, AWY10427.1, AXY82632.1, AJT61420.1, AJT61311.1, YP_009055463.1, AID17956.1, QBY34658.1, QBY34659.1, QBY34660.1 and QBY34657.1	https://swissmodel.expasy.org/templates/6et6.1.A
KU510289.1, NC_041857.1, MH853786.1, AJG41880.1, AJG41879.1, AJG41878.1, AJG41877.1 and vB_AbaM-SHI	https://swissmodel.expasy.org/templates/2nr7.1.A
ALJ97637.1, ASN73403.1, ASN73457.1 and ASN73506.1	https://swissmodel.expasy.org/templates/2z38.1.A
AJG41885.1, AJG41884.1, AJG41883.1 and AJG41882.1	https://swissmodel.expasy.org/templates/3pbi.1.A

### 2.11 Statistical analyses

Statistical significance was analyzed using SPSS statistical software (ver. 11.5). The killing activity of phage vB_AbaM-SHI in the experiments were expressed as logarithmic reduction ± standard error (Zelver et al., 2001). Multiple t tests were performed to determine differences between groups with a *P* value of < 0.05 considered significant (*, *P <*0.05; **, *P <*0.01; ***, *P <*0.001).

## 3 Results

### 3.1 Biological characteristics of phage vB_AbaM-SHI

**Figure 1:**
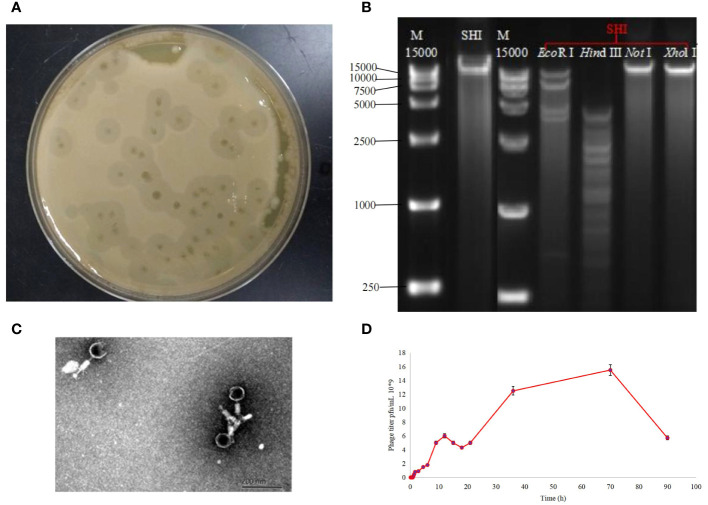
Biological characteristics of phage vB_AbaM-SHI. **(A)** Bacteriolytic plaques appearing on *A. baumannii* plates inoculated with phage vB_AbaM-SHI; **(B)** Phage vB_AbaM-SHI genomic DNA was cleaved by restriction endonucleases EcoRI and Hind III (lanes 4 and 5), but not by NotI and Xhol I (lanes 6 and 7); **(C)** Morphology of phage vB_AbaM-SHI under transmission electron microscopy (TEM); **(D)** Growth curve of phage vB_AbaM-SHI propagated in *A. baumannii* SL. “M”: DNA marker; “SHI”: vB_AbaM-SHI.

Using *A. baumannii* as host bacterium, we isolated a bacteriophage (named as *A. baumannii* phage vB_AbaM-SHI) from a sauce chop factory drain outlet in Wuxi, Jiangsu, China. Phage vB_AbaM-SHI formed the plaques with around 5 mm in diameter (**Figure 1A**). Phage vB_AbaM-SHI DNA was cleaved by restriction endonucleases EcoRI and Hind III, but not by Xhol I and NotI (**Figure 1B**). TEM images showed that phage vB_AbaM-SHI virions have a tail with a length of around 30 nm and an icosahedral head with an estimated diameter of 60 nm (**Figure 1C**). It was estimated from the growth curve of phage vB_AbaM-SHI in *A. baumannii* that the latent period of phage vB_AbaM-SHI is about 50 min, and the burst size of phage vB_AbaM-SHI is approximately 155 pfu/cell. The titers of phage vB_AbaM-SHI increased very quickly from the 4.5 h after initial phage infection, and reached the peak at the 12 h post phage infection. In addition, phage vB_AbaM-SHI has an amplification factor of approximately 5000 times (**Figure 1D**).

**Figure 2:**
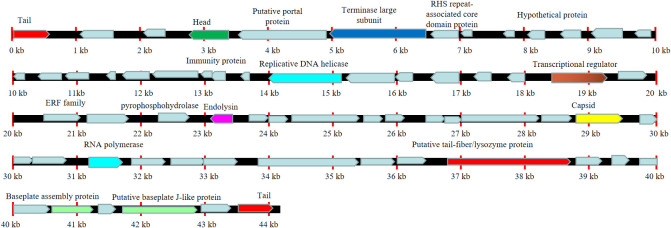
Line map of the *A. baumannii* phage vB_AbaM-SHI genome. In the vB_AbaM-SHI genome track, genes colored in red encode tail and genes colored in green encode head. The arrows represent the ORFs and point in the direction of transcription. Replicative DNA helicase and RNA polymerase were marked with cyan rectangular box, cell lysis was marked with purple rectangular box, baseplate assembly proteins were marked with light green rectangular box, and capsid was marked with yellow rectangular box.

**Figure 3:**
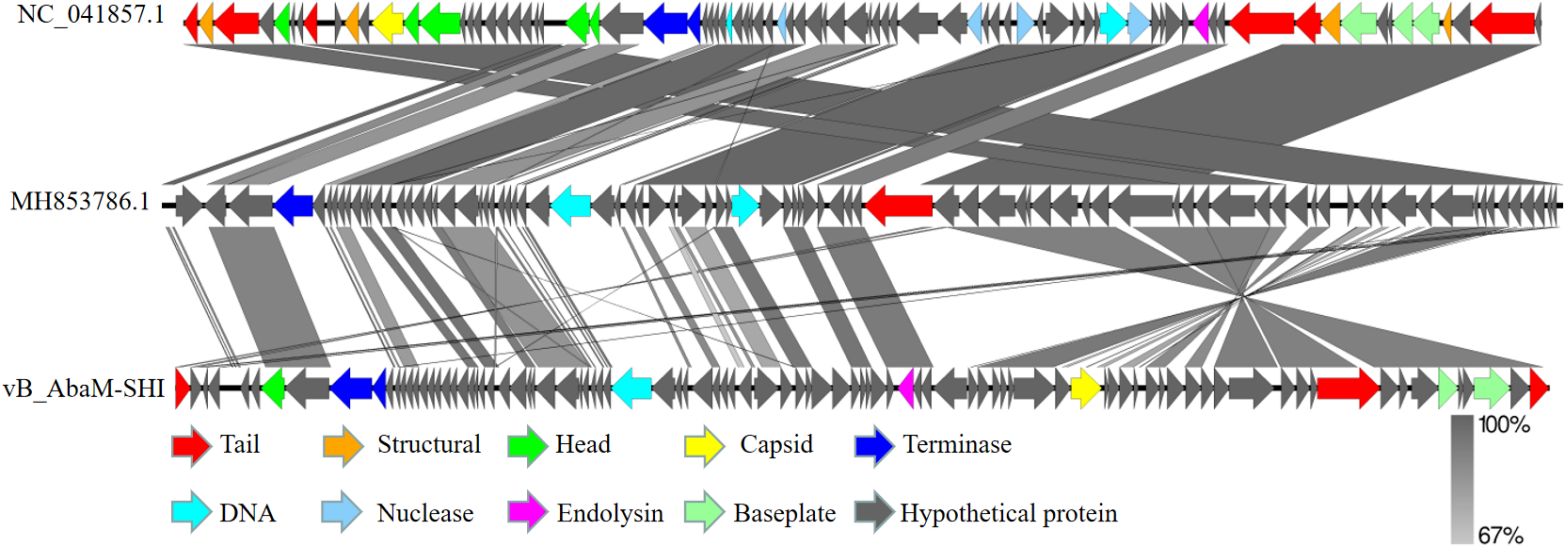
Easyfig output image of the genomic comparison among *Acinetobacter* phage IME-AB2 (top, NC_041857.1), *Acinetobacter* phage vB_AbaM_IME285 (middle, MH853786.1) and *Acinetobacter* phage vB_AbaM-SHI (bottom). Phage genomes are presented by linear visualization with coding regions shown as arrows. ORFs are color-coded according to predicted function: red, tail; orange, structural; green, head; yellow, capsid; blue, terminase; purple, endolysin; grey, hypothetical proteins. The percentage of sequence similarity is indicated by the intensity of the gray color. Vertical blocks between analyzed sequences indicate regions with at least 67% of similarity.

We next sent the extracted nucleic acid of phage vB_AbaM-SHI to a company for sequencing. The results showed that this phage genome contains 44,180 bp, with G+C contents of 38.14%. PCR amplification on primers targeting both ends of the genome did not yield products, suggesting that the phage genome should be linear. We identified 93 open reading frames (ORFs) in the complete genome of vB_AbaM-SHI (**Figure 2**; **Supplementary Table S1**). Among them, 18 ORFs encode functional proteins which can be categorized into five groups: DNA packaging and nucleic acid metabolism, cell lysis, structure proteins except tail fiber, tail-associated protein and hypothetical protein (**Figure 2**; **Supplementary Table S1**). The remaining 75 ORFs encode hypothetical proteins with unknown functions (**Supplementary Table S1**). In addition, no homologous sequences highly similar to published antimicrobial resistant genes (ARGs) or phage virulence factors were identified in the genome of phage vB_AbaM-SHI. Based on these facts, namely that this phage can efficiently lyse host bacteria, and there is a lack of integrase and transposon genes related to the entry of the phage into the lysogenic state in the genome of phage vB_AbaM-SHI, we carefully speculate that this phage should belong to lytic phage rather than lysogenic phage. The whole-genome alignment shows that vB_AbaM-SHI is most similar to *Acinetobacter* phage IME-AB2 and *Acinetobacter* phage vB_AbaM_IME285 in terms of genomes and module conformation (**Figure 3**). These three phages have more than 50% nucleotide identity, similar tRNA numbers, and similar G + C% in the genome. Therefore, according to the current criteria of the International Committee on Taxonomy of Viruses (ICTV) (Peng et al., 2014; Wang et al., 2020b), phage vB_AbaM_IME285 can be classified as family *Straboviridae*.

### 3.2 Optimum pH, MOI and temperature for the proliferation of phage vB_AbaM-SHI

**Figure 4:**
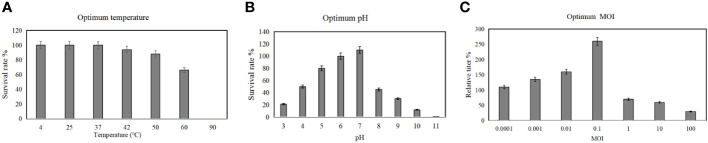
Optimum temperature, pH and MOI for the replication of phage vB_AbaM-SHI. **(A)** Stability of phage vB_AbaM-SHI at different temperatures; **(B)** Stability of vB_AbaM-SHI at different pH levels; **(C)** Optimal multiplicity of infection (MOI) determination.

Temperature, pH of culture medium and MOI are critical factors affecting the replication efficiency of phages, and these factors also potentially affect the bacteriolytic effect of phages. Therefore, to determine the stability of phage vB_AbaM-SHI at various temperatures and pH levels, we added phage to buffers with different pH levels or incubated them in different temperature environments, and then measured the titers of phages in the liquid. This phage exhibited a good resistance to temperatures ranging from 4°C to 37°C, and there is no significant difference in the titer of phages within this temperature range. In comparison, the titer of phages at 60°C was significantly reduced and the phages were completely inactivated at 90°C (**Figure 4A**). The most proper pH values to maintain phage activity ranged from 6 to 7, however, the activity of phages was significantly reduced when the phages were exposed to alkaline buffer solutions with pH 10 or pH 11 (**Figure 4B**). Overall, phage vB_AbaM-SHI has a high tolerance to acidic environments, as about 20% of phages remain infectious after being exposed to a pH 3 buffer for one hour, while almost all phages lost their infectivity after being exposed to a pH 11 buffer for one hour. When using bacteria to amplify phage vB_AbaM-SHI, the production of phages is inversely proportional to the MOI value during phage infection. As the MOI value increased from 0.1 to 100, the production of phages gradually decreased. When the MOI value is 0.1, the yield of phages reached the highest (**Figure 4C**).

### 3.3 Phage vB_AbaM-SHI has broad-spectrum bacteriolytic ability

Before testing the ability of phage vB_AbaM-SHI to lyse clinically isolated *A. baumannii* strains, we first determine the sensitivity of *A. baumannii* strains to antibiotics. As a result, seven *A. baumannii* strains exhibited different sensitivities to 5 antibiotics tested. Among them, more than half (5/7) of the bacterial strains had resistance to 3 antibiotics, approximately half (3/7) of the bacterial strains demonstrated sensitivity to tobramycin, and all bacterial strains were sensitive to colistin (**Table 3**). We next tested the sensitivity of 7 A*. baumannii* strains and 4 other bacterial strains to phage vB_AbaM-SHI. The results showed that approximately half of (3/7) of *A. baumannii* strains could be lysed by the phage (the diameters of lysis zone ranging from 5 to 7 mm). In addition, *E. coli* BL21 strain was also lysed by this phage (with a lysis zone of 6 mm in diameter). However, both *S. aureus* strain and *S. paratyphi* strains were not sensitive to this phage lysis (without visible lysis zone) (**Table 4**).

**Table 3:** Resistance of *A. baumannii* strains to antibiotics.

Antibiotics	*A. baumannii* strains *^a^*						
	SL	SL1	SJ9	SA5	SA3	SG	SH
Ampicillin	R	R	R	R	S	S	R
Ciprofloxacin	R	R	R	R	S	S	R
Levofloxacin	R	R	R	R	S	S	R
Tobramycin	R	R	R	S	S	S	R
Colistin	S	S	S	S	S	S	S

a R, resistance; S, sensitivity. SL, SL1, SJ9, SA5, SA3, SG and SH represent strains of A baumannii.

**Table 4:** Sensitivity of *bacterial* strains to phage vB_AbaM-SHI.

Bacterial strains	Samples	Source of bacteria	Sensitivity to vB_AbaM-SHI^a^
*S. aureus* cpx	Fecal smaples	(Jiang et al., 2021a)	–
*S. paratyphi A-B*	Fecal smaples	(Jiang et al., 2021b)	–
*E. coli BL21*	No pathogenic	TransGen Biotech	+
*S. paratyphi* A NA3	Fecal smaples	(Jiang et al., 2021b)	–
*A. baumannii* SL	Sputum	CMCPC	+
*A. baumannii* SL1	Sputum	This study	+
*A. baumannii* SJ9	Sputum	This study	–
*A. baumannii* SA5	Sputum	This study	–
*A. baumannii* SA3	Sputum	This study	–
*A. baumannii* SG	Sputum	This study	+
*A. baumannii* SH	Sputum	This study	–

a , +clear lysis zone; -no lysis zone.

### 3.4 Phage can independently or in combination with antibiotics inhibit the formation of bacterial biofilms

**Figure 5:**
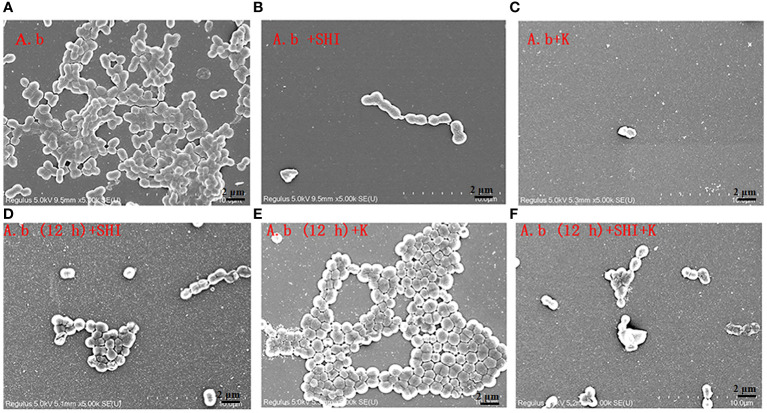
Scanning electron microscopy (SEM) image of *A. baumannii* SL colonization in biofilms formed on a round coverslip before and after phage vB_AbaM-SHI and kanamycin sulfate treatment. **(A)** *A. baumannii* SL was diluted in the overnight culture (1:250) and cultured for 24 hours before the addition of **(B)** phage vB_AbaM-SHI (MOI = 0.1) or **(C)** kanamycin sulfate (10 µg mL^-1^); **(D)** *A. baumannii* SL was diluted in the overnight culture (1:250) and cultured for 12 hours, and then phage vB_AbaM-SHI (MOI = 0.1) was added and cultured for another 12 hours; **(E)** *A. baumannii* SL was diluted in the overnight culture (1:250) and cultured for 12 hours, and then kanamycin sulfate (10 µg mL^-1^) was added and cultured for another 12 hours; **(F)** *A. baumannii* SL was diluted in the overnight culture (1:250) and cultured for 12 hours, and then both phage vB_AbaM-SHI (MOI = 0.1) and kanamycin sulfate (10 µg mL^-1^) were added and cultured for another 12 hours. The colonization of bacteria on coverslip was analyzed using scanning electron microscopy (5, 000× magnification). “A.b”: *A. baumannii*; “SHI”: vB_AbaM-SHI; “K”: kanamycin sulfate.

**Figure 6:**
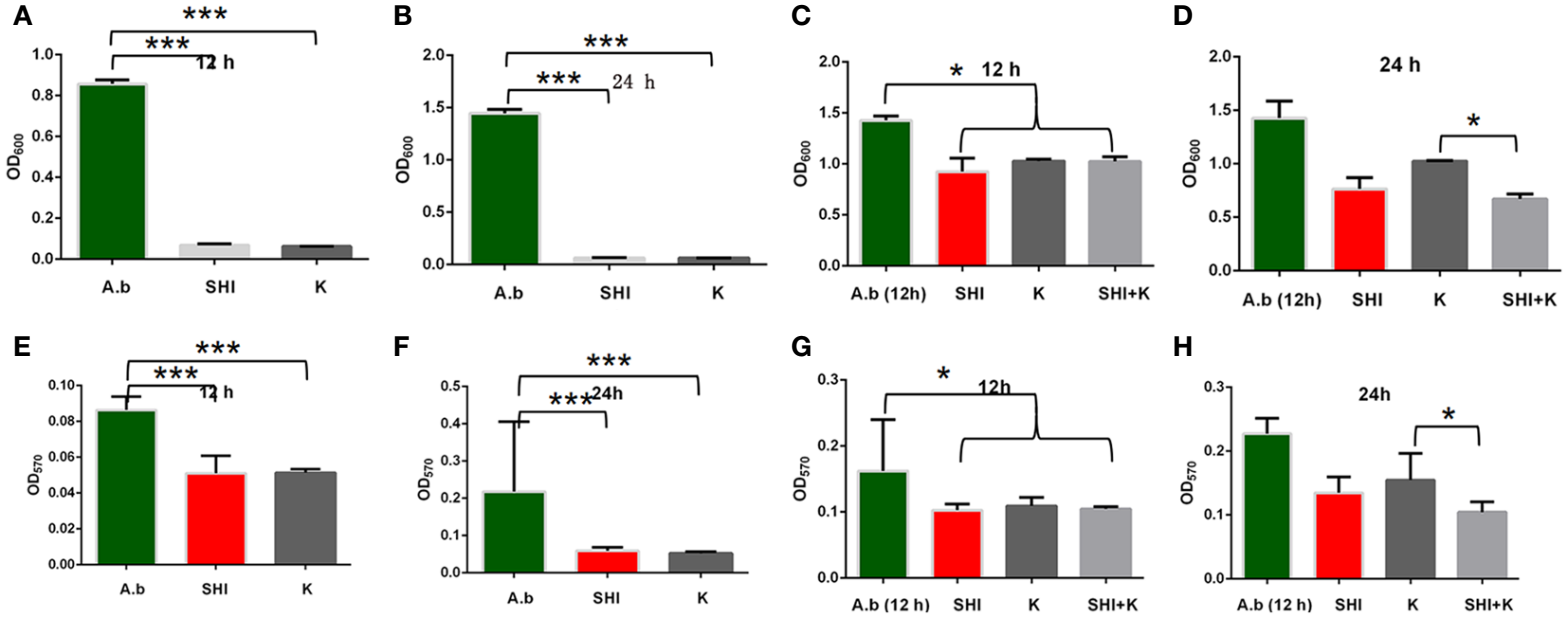
Effects of phage vB_AbaM-SHI and kanamycin sulfate on planktonic and bacterial biofilm formation. **(A, B)** Effects of phage vB_AbaM-SHI and kanamycin sulfate (10 µg mL^-1^) on the growth of *A. baumannii* SL after pre-cultivation for 12 h **(A)** and 24 h **(B)**. **(C, D)** Effects of phage vB_AbaM-SHI and kanamycin sulfate (10 µg mL^-1^) on *A. baumannii* SL (inoculated at a concentration of 4‰) growth after first being cultured for 12 h, followed by phage *A. baumannii* SL and kanamycin sulfate (10 µg mL^-1^) addition and culturing for 12 h **(C)** and 24 h **(D)**. **(E, F)** Effects of phage vB_AbaM-SHI and kanamycin sulfate (10 µg mL^-1^) on *A. baumannii* SL (inoculated at a concentration of 4‰) biofilm formation after pre-cultivation for 12 h **(E)** and 24 h **(F)**. **(G, H)** Effects of phage vB_AbaM-SHI and kanamycin sulfate (10 µg mL^-1^) on *A. baumannii* SL (inoculated at a concentration of 4‰) biofilm formation after an initial culture for 12 h, followed by the addition of phage vB_AbaM-SHI and kanamycin sulfate (10 µg mL^-1^) and culture for 12 **(G)** and 24 h **(H)**. *P <0.05, ***P <0.001. “A.b”: *A. baumannii*; “SHI”: vB_AbaM-SHI; “K”: kanamycin sulfate.

**Figure 7:**
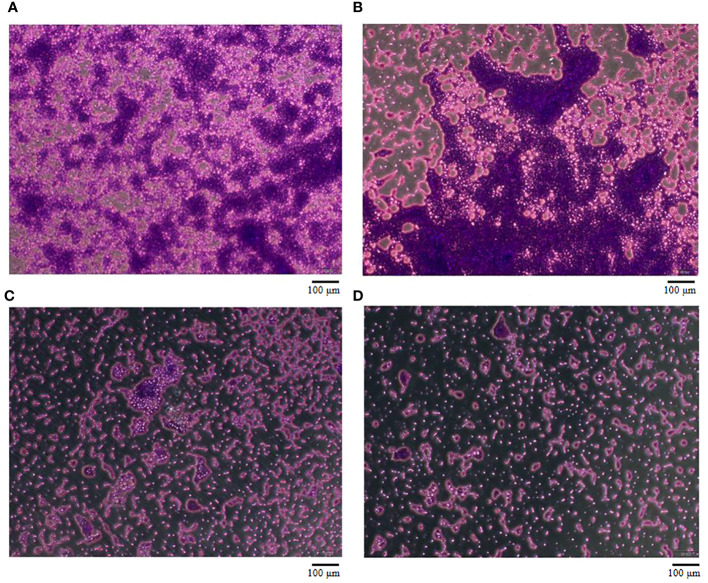
The effect of Phage vB_AbaM-SHI on the biofilm formation of *A. baumannii* SL. **(A-C)** Biofilm formed by *A. baumannii* SL at 24 h, 36 h and 60 h, respectively. **(D)** Phage vB_AbaM-SHI inhibited the formation of *A. baumannii* SL biofilm (MOI = 1, 36 h).

**Figure 8:**
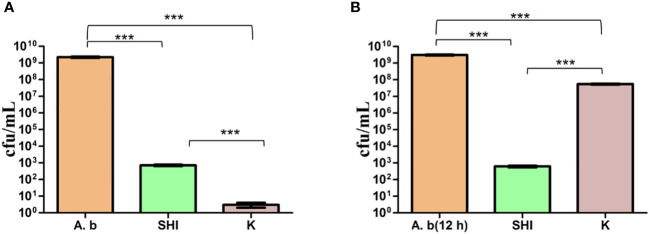
Phage vB_AbaM-SHI and kanamycin sulfate inhibit the growth of *A. baumannii* SL. **(A)** Effects of phage vB_AbaM-SHI and kanamycin sulfate (10 µg mL^-1^) on the growth of *A. baumannii* SL (diluted in the overnight culture [1:250]) cultured for 24 h. **(B)** Effects of phage vB_AbaM-SHI and kanamycin sulfate (10 µg mL^-1^) on *A. baumannii* SL (diluted in the overnight culture [1:250]) after an initial culture for 12 hours followed by the addition of phage vB_AbaM-SHI and kanamycin sulfate (10 µg mL^-1^) and culture for 12 h. ***P <0.001. “A.b”: *A. baumannii*; “SHI”: vB_AbaM-SHI; “K”: kanamycin sulfate.

To evaluate the effect of vB_AbaM-SHI (MOI = 0.1) and kanamycin sulfate (10 µg mL^-1^) on the biofilm formation of bacteria, we conducted inhibition experiments of bacterial growth in 96-well plates and the OD_570_ or OD_600_ (optical density at 570 nm or 600 nm wavelength) values of each well were measured at 12 h and 24 h post bacterial inoculation. When *A. baumannii* SL cells were in a low-density state, all results from scanning electron micrographs (**Figures 5A–C**; reflecting the amount of bacterial biofilm at the bottom of the cell culture plate), OD_600_ values (**Figures 6A**; reflecting the amount of bacteria in the suspension), and OD_570_ values (**Figures 6E, F**; reflecting the amount of bacterial biofilms at the bottom of the cell culture plate) indicated that phage vB_AbaM-SHI and kanamycin sulfate has similar antibacterial effects. In contrast, when *A. baumannii* SL cells were locked in the high-density state, phage vB_AbaM-SHI exerted a better antibacterial effect than kanamycin sulfate (**Figures 5D–F**). In addition, compared to the ability to inhibit biofilm formation, phage vB_AbaM-SHI had a strong ability to control the growth of suspended cells (**Figure 6**). We then evaluated the ability of phage vB_AbaM-SH and kanamycin sulfate to inhibit the bacterial biofilm formation. The biofilm biomass of *A. baumannii* SL was measured using epifluorescence microscopy at 24 h, 36 h and 60 h post bacterial inoculation, respectively. The results showed that the amount of biofilm reached the peak at 36 h. Phage vB_AbaM-SHI significantly and persistently inhibited the formation of bacterial biofilm for up to 36 h (**Figure 7**). After adding phage vB_AbaM-SHI or kanamycin sulfate to *A. baumannii* and culturing for 24 hours, the amount of bacterial biofilm was measured. Both treatment factors can significantly inhibit the formation of bacterial biofilm, and kanamycin sulfate is superior to phage vB_AbaM-SHI (**Figure 8A**). Then, we first cultured the bacteria for 12 hours before adding phage vB_AbaM-SHI or kanamycin sulfate and determined the amount of bacterial biofilm. Although both can significantly reduce the amount of bacterial biofilm, phage vB_AbaM-SHI is significantly better than kanamycin sulfate (**Figure 8B**). In summary, phage vB_AbaM-SHI and kanamycin sulfate have distinct characteristics in inhibiting bacterial growth and controlling bacterial biofilm formation.

### 3.5 Structure and molecular specificity of vB_AbaM-SHI endolysin

Phage endolysin is a cell wall hydrolase that is encoded by a phage gene and synthesized at the late stage of phage infection (Fischetti, 2008). In general, endolysin needs to work in synergy with holin or spanin protein to lyse gram-positive or gram-negative bacteria, respectively (Singh et al., 2022). By comparing the genome sequence of phage vB_AbaM-SHI with database, we found that there is a lack of genes encoding holin or spanin in phage vB_AbaM-SHI, suggesting endolysin in phage vB_AbaM-SHI could be able to exert bacteriolytic effect alone. In fact, although it is difficult for endolysin alone to penetrate the outer membrane of gram-negative bacteria and destroy the peptidoglycans in bacterial cell wall, recent studies reported that some phage endolysins can independently lyse gram-negative bacteria. For instance, the recombinant endolysin LysSS exhibited bacteriolytic activity against *A. baumannii*, *E. coli*, *K. pneumoniae*, *Salmonella* sp. and *P. aeruginosa* without pre-treatment with an outer membrane permeabilizer (Kim et al., 2020). Similarly, another *A. baumannii* phage endolysin LysAB54 with high antibacterial activity against multiple multidrug-resistant *A. baumannii* and other gram-negative bacteria, including *E. coli*, *P. aeruginosa* and *K. pneumoniae*, in the absence of outer membrane permeabilizer (Khan et al., 2021). Moreover, a recent study reported a novel lysis mechanism that *Salmonella* endolysin M4Lys exerted its bacteriolytic activity, which was not dependent on either holin or the Sec pathway (Bai et al., 2020). Considering that endolysin may be an independent factor for phage vB_AbaM-SHI to exert bacteriolytic effect, we next compared and analyzed the phylogenetic relationship between our endolysin and similar endolysins. In general, the endolysins of *A. baumannii* phages have significant differences in size, amino acid sequence, and homology. The lengths of KU510289.1, NC_041857.1, AJG41879.1, AJG41878.1, ASN73457.1 AJG41884.1, YP_004009630.1 AWY10427.1 AID17956.1, QBY34659.1 and QBY34657.1 range from 145 to 256 amino acids (**Supplementary Figure S1**; **Table 1**). The tertiary structures predicted using SWISS-MODEL (https://swissmodel.expasy.org/) showed that these endolysins share common modeling template (**Table 2**). *A. baumannii* phage endolysins with similar structures are located in very close branch of evolution, for example, KU510289.1, NC_ 041857.1, MH853786.1, and vB_AbaM-SHI are in the same evolutionary branch (**Supplementary Figure S1**). The structures of *A. baumannii* phage endolysins demonstrated significant differences in β strands and α helices (**Supplementary Figure S1**).

**Figure 9:**
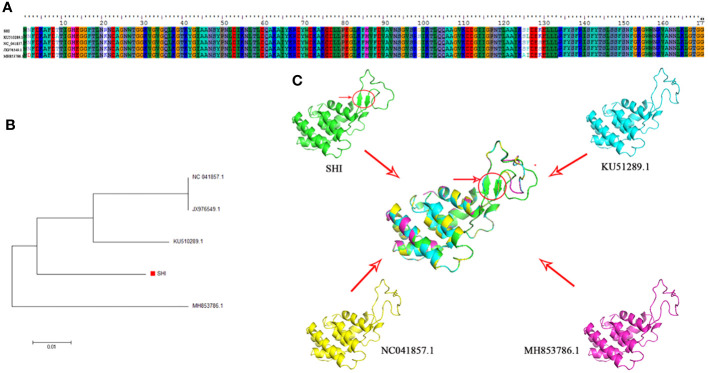
Amino acid sequence alignment, phylogenetic analysis and predicted tertiary structure of *A. baumannii* phage endolysins **(A)** Amino acid sequence alignment of endolysin proteins among vB_AbaM-SHI, KU510289.1, NC_041857.1, and MH853786.1. **(B)** Phylogenetic tree of *A. baumannii* phage endolysins. (The red square denotes the *A. baumannii* phage vB_AbaM-SHI endolysin). **(C)** Predicted tertiary structure of the *A. baumannii* phage endolysin protein. Superposition of vB_AbaM-SHI (green), KU510289.1 (cyano), NC_041857.1 (yellow), and MH853786.1 (purple) structures (https://swissmodel.expasy.org/templates/2nr7.1), which was reproduced/adapted from Structural Genomics, the crystal structure of putative secretion activator protein from Porphyromonas gingivalis W83, by Tan, K., Bigelow, L., Gu, M., Joachimiak, A., Midwest Center for Structural Genomics (MCSG), https://swissmodel.expasy.org/templates/2nr7.1, licensed CC-BY-SA-4.0. “SHI”: vB_AbaM-SHI.

By comparing the amino acid sequences of endolysins, it can be found that KU510289.1, NC_041857.1, JX976549.1, vB_AbaM-SHI and MH853786.1 have amino acid sequence differences mainly at positions 2, 9, 21, 99, 125, 126 and 129 (**Figure 9**). Intriguingly, we identified that phage vB_AbaM-SHI endolysin contains two additional β strands, attributing to the amino acid change at position 21 [lysine (K) in KU510289.1, NC_041857.1, JX976549.1 and MH853786.1 was replaced with arginine (R) in vB_AbaM-SHI].

## 4 Discussion

Lytic phages were usually isolated from sewage (Carey-Smith et al., 2006). In the present study, phage vB_AbaM-SHI was obtained from sauce chop factory drain outlet samples in Wuxi, China and classified as the family *Straboviridae.* Phage vB_AbaM-SHI has a genome with 44,180 bp and G+C contents of 38.14%. In comparison, the genomes of *A. baumannii* phage vB-GEC_Ab-M-G7 and phage BΦ62, two members of family *Straboviridae*, are 90 kb and 44,844 bp, respectively (Jeon et al., 2016; Kusradze et al., 2016). In addition, 93 ORFs were identified in the genome of vB_AbaM-SHI, whereas phage 5W has 61 putative ORFs in the genome (Peng et al., 2021). The phylogenetic analysis showed that phage vB_AbaM-SHI endolysin has high amino acid sequence homology with endolysins described in previous studies. No antibiotic resistance and virulence associated genes were identified in vB_AbaM-SHI genome.

With the increase in antibiotic misused, the emergence of multidrug-resistant and extremely resistant bacteria has become a serious threat to human health. Alternatives to antibiotics are urgently needed. Hopefully, phages represent an alternative solution to the issue of drug resistance. Although vB_AbaM-SHI barely lysed bacteria from other genera, vB_AbaM-SHI exhibited broad-spectrum bacteriolytic activity against multidrug-resistant *A. baumannii* that were resistant to one or more commonly used antibiotics. Lytic phages usually show a narrow lytic spectrum. Different bacterial strains have distinct differences in the sensitivity to the phage, which restrict the potential application of phage. For example, Wintachai et al., reported that phage vABWU2101 has the ability to kill 70% of the tested MDR *A. baumanni*i strains (Wintachai et al., 2022). In contrast, Peng et al., demonstrated that phage 5W only lyse 21% *A. baumannii* isolates (Peng et al., 2021). Herein, phage vB_AbaM-SHI could lyse 42.8% of *A. baumannii* strains which were isolated from clinical patients, suggesting that vB_AbaM-SHI has the potential to be used for the treatment of corresponding clinical bacterial infections.

Biofilm is usually defined as a surface-attached microbial community, which mainly protects bacteria from the external dangerous environment (Qi et al., 2016). The ability of bacteria to form biofilms on different surfaces increases the risk of contamination, particularly in poultry products, clinics and food industries (Liu et al., 2019; Lianou et al., 2020). Although the exploitation of treatments that interrupt multidrug efflux pumps and quorum sensing interference strategies are important for inhibiting biofilm formation (Subhadra et al., 2018), developing effective strategies to eliminate biofilms remains a challenge and ideal agents for bacteria biofilm control are not available (Joshi et al., 2021; Li et al., 2021). The biofilm formation of *A. baumannii* was affected by the phenotype, genotype, physicochemical characteristics of bacteria (Eze et al., 2018; Yang et al., 2019). In the present study, kanamycin sulfate displayed good inhibiting ability of bacterial biofilm formation in low-density *A. baumannii* SL culture. But, phage vB_AbaM-SHI exerted better anti-biofilm formation and sterilization effects than kanamycin sulfate in a high-density *A. baumannii* SL culture. Additionally, the combination of phage vB_AbaM-SHI and kanamycin sulfate displayed strong anti-biofilm formation and antibacterial effects than phage vB_AbaM-SHI or kanamycin sulfate alone in a low-density *A. baumannii* SL culture. However, compared to killing suspended *A. baumannii* SL strain, phage vB_AbaM-SHI has weaker ability to lyse bacteria in biofilms, suggesting this phage has weak penetrating ability. Therefore, reagents or strategies that help phages penetrate biofilms are with great significance. Taken together, phage vB_AbaM-SHI is expected to become a promising agent to control multidrug-resistant *A. baumannii*. In fact, Grygorcewicz et al., recently revealed that a *A. baumannii* phages cocktail in combination with antibiotics had strong lytic ability to destroy biofilm in human urine (Grygorcewicz et al., 2021).

In summary, we identified a broad-spectrum phage vB_AbaM-SHI that exhibited a potent lytic effect on *A. baumannii* strains isolated in clinic. vB_AbaM-SHI can independently kill bacteria and inhibit bacterial biofilm formation, and can also synergistically exert strong antibacterial effects with antibitics, indicating vB_AbaM-SHI is a promising agent for the bio-control of multi-drug resistant *A. baumannii* strains and preventing the biofilm formation on medical devices.

## Data availability statement

The datasets presented in this study can be found in online repositories. The names of the repository/repositories and accession number(s) can be found below: ON480525.1.

## Author contributions

LJ: Conceptualization, Writing – original draft, Writing – review & editing. QX: Writing – original draft. YW: Formal analysis, Writing – review & editing. XZ: Resources, Conceptualization, Formal analysis, Validation, Writing – original draft. ZC: Methodology, Writing – original draft. QS: Software, Writing – original draft. JW: Conceptualization, Supervision, Writing – review & editing.

## Supplementary material

Supplementary Figure 1 The characteristics of *A. baumannii* phage endolysin. a, Amino acid substitutions of *A. baumannii* phage endolysin proteins among vB_AbaM-SHI **(A)**, AJG41880.1 **(B)**, ALJ97637.1 **(C)**, AJG41885.1 **(D)**, ADG35978.1 **(E)**, AXF40585.1 **(F)**, AJT61420.1 **(G)** and QBY34658.1 **(H)**. b, Phylogenetic tree of *A. baumannii* phage endolysin proteins among vB_AbaM-SHI **(A)**, AJG41880.1 **(B)**, ALJ97637.1 **(C)**, AJG41885.1 **(D)**, ADG35978.1 **(E)**, AXF40585.1 **(F)**, AJT61420.1 **(G)** and QBY34658.1 **(H)**. **(C)**, Predicted tertiary structures of *A. baumannii phage* endolysin proteins among vB_AbaM-SHI **(A)**, AJG41880.1 **(B)**, ALJ97637.1 **(C)**, AJG41885.1 **(D)**, ADG35978.1 **(E)**, AXF40585.1 **(F)**, AJT61420.1 **(G)** and QBY34658.1 **(H)**.
