# Supplementary figures and images for: Characterization of a Straboviridae phage vB_AbaM-SHI and its inhibition effect on biofilms of Acinetobacter baumannii
Source: Front Cell Infect Microbiol. 2024 Mar 8;14:1351993. doi: 10.3389/fcimb.2024.1351993 (PMC10958429; doi:10.3389/fcimb.2024.1351993)

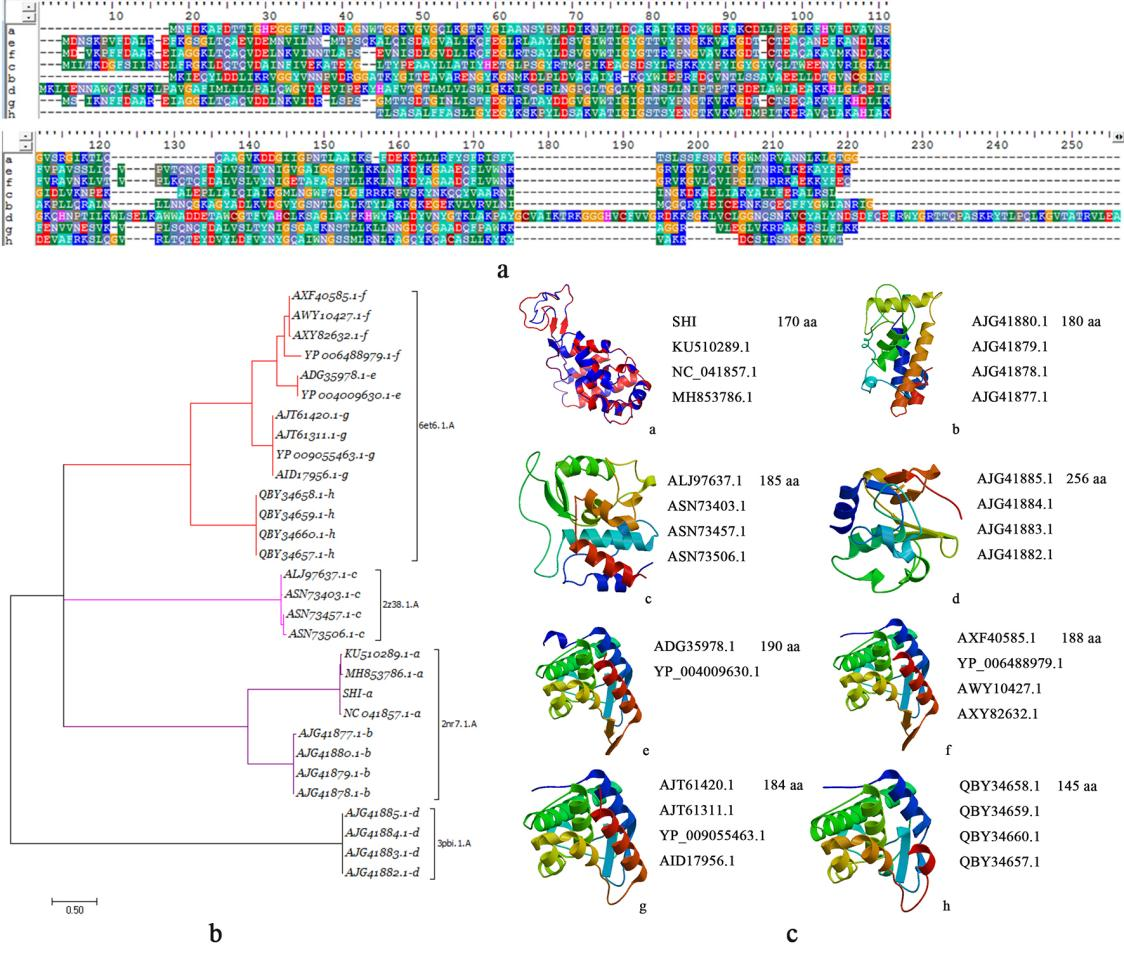

Supplement: Supplementary Figure 1 — The characteristics of A. baumannii phage endolysin. a, Amino acid substitutions of A. baumannii phage endolysin proteins among vB_AbaM-SHI (A), AJG41880.1 (B), ALJ97637.1 (C), AJG41885.1 (D), ADG35978.1 (E), AXF40585.1 (F), AJT61420.1 (G) and QBY34658.1 (H). b, Phylogenetic tree of A. baumannii phage endolysin proteins among vB_AbaM-SHI (A), AJG41880.1 (B), ALJ97637.1 (C), AJG41885.1 (D), ADG35978.1 (E), AXF40585.1 (F), AJT61420.1 (G) and QBY34658.1 (H). (C), Predicted tertiary structures of A. baumannii phage endolysin proteins among vB_AbaM-SHI (A), AJG41880.1 (B), ALJ97637.1 (C), AJG41885.1 (D), ADG35978.1 (E), AXF40585.1 (F), AJT61420.1 (G) and QBY34658.1 (H). [file Image_1.jpeg]
